# Current status and issues in genomic analysis using EUS-FNA/FNB specimens in hepatobiliary–pancreatic cancers

**DOI:** 10.1007/s00535-023-02037-z

**Published:** 2023-09-12

**Authors:** Yoshinori Ozono, Hiroshi Kawakami, Naomi Uchiyama, Hiroshi Hatada, Souichiro Ogawa

**Affiliations:** Division of Gastroenterology and Hepatology, Department of Internal Medicine, Faculty of Medicine, University of Miyazaki, 5200 Kihara, Kiyotake, Miyazaki, 889–1692 Japan

**Keywords:** Comprehensive genomic profiling, Endoscopic ultrasound, EUS-guided fine-needle aspiration, EUS-guided fine-needle biopsy, Hepatobiliary–pancreatic cancers

## Abstract

Comprehensive genomic profiling based on next-generation sequencing has recently been used to provide precision medicine for various advanced cancers. Endoscopic ultrasound (EUS)-guided fine-needle aspiration (EUS-FNA) and EUS-guided fine-needle biopsy (EUS-FNB) play essential roles in the diagnosis of abdominal masses, mainly pancreatic cancers. In recent years, CGP analysis using EUS-FNA/FNB specimens for hepatobiliary–pancreatic cancers has increased; however, the success rate of CGP analysis is not clinically satisfactory, and many issues need to be resolved to improve the success rate of CGP analysis. In this article, we review the transition from EUS-FNA to FNB, compare each test, and discuss the current status and issues in genomic analysis of hepatobiliary–pancreatic cancers using EUS-FNA/FNB specimens.

## Introduction

Endoscopic ultrasound (EUS)-guided fine-needle aspiration (EUS-FNA), a tissue sampling method using EUS, is a well-established method for the pathological diagnosis of pancreatic and gastrointestinal submucosal tumors, as well as lymph node lesions [1]. Particularly for pancreatic tumors, EUS-FNA is a highly accurate diagnostic method with a sensitivity and specificity of over 90% [2]. However, with the recent development of immune therapy for malignant tumors, individualized treatments based on genetic mutations have been provided in daily practice. Now, quantity and quality are required for specimens that can withstand genetic testing and diagnosis.

The usefulness of rapid on-site evaluation (ROSE) has long been reported because of the difficulty in pathological diagnosis due to the small volume of specimens obtained by EUS-FNA. Specimens are processed through cytology in facilities that can perform ROSE, whereas histology is used in facilities where ROSE is cannot to perform [3]. Subsequently, histological examinations are usually performed to improve diagnostic adequacy, and EUS-guided fine-needle biopsy (EUS-FNB) has become popular in recent years. It has been reported that EUS-FNB has a high diagnostic accuracy without ROSE because a larger volume of specimens can be collected than with EUS-FNA [4].

In addition, the quality and the quantity of EUS-FNB specimens have shown better potential than those of EUS-FNA specimens, and the usefulness of EUS-FNB in genetic testing has been increasingly reported [5].

Comprehensive genomic profiling (CGP) is a testing method that uses next-generation sequencing (NGS) to analyze a large amount of genomic information comprehensively, attracting considerable attention because of its ability to detect genetic abnormalities that may lead to genome-matched therapy. Since 2019, CGP testing, OncoGuide™ NCC Oncopanel System (NOP; Sysmex Corporation, Hyogo, Japan), and FoundationOne^®^ CDx (F1CDx; Foundation Medicine, Cambridge, MA) have been covered by national health insurance in Japan for solid cancers that are un-resectable and refractory to standard therapies. However, the feasibility, optimal needle selection, and the number of punctures required for EUS-FNA/FNB have not yet been clarified. In this review, we discuss the transition from EUS-FNA to FNB, compare these methods, and discuss the current status and issues in genetic analysis using EUS-FNA/FNB specimens for hepatobiliary–pancreatic cancers.

## Comparison of tissue sampling methods by EUS-FNA and EUS-FNB

### Diagnostic accuracy

Since 2010, pathological diagnosis using EUS-FNA for abdominal mass lesions, mainly pancreatic cancers, has been performed in Japan and is now a widely performed procedure. In particular, the diagnostic accuracy of EUS-FNA for pancreatic tumors has been reported in a meta-analysis to achieve a sensitivity of 84–92%, specificity of 96–98%, and diagnostic accuracy rate of 86–91%, proving its effectiveness [2, 6, 7]. The needle sizes for EUS-FNA range from 19 to 25-gage; a 25-gage fine needle is particularly maneuverable for duodenal manipulation in diagnosing benign and malignant diseases. Madhoun et al. reported that a 25-gage needle was more sensitive than a 22-gage needle for pancreatic tumors [8].

Precision medicine, in which mutant genes are comprehensively analyzed and applied to the individualized treatment of various advanced solid cancers, has gained popularity in recent years; accordingly, the quantity and the quality of specimens have become more critical than before. Subsequently, the core biopsy needle was developed to obtain more tissue samples, and EUS-FNB was clinically performed for histological diagnosis. The core-trap, Franseen, and fork-tip needles are well-known representative needles that can be used in Japan. Negative-pressure methods for tissue sampling during EUS-FNA/FNB include syringe aspiration, non-aspiration, and slow-pull methods, in which the stylet is slowly pulled out. A meta-analysis comparing the aspiration and slow-pull methods during EUS-FNA/FNB for pancreatic tumors showed less blood contamination with the slow-pull method; however, the results were controversial and inconclusive regarding the diagnostic accuracy rate [9–11]. Prior to the introduction of EUS-FNA in the 1990s, pancreatic cancers were diagnosed using endoscopic retrograde cholangiopancreatography (ERCP), which has a low sensitivity (49–66%) and a high complication rate, including post-ERCP pancreatitis [12]. EUS-FNA/FNB has a higher diagnostic accuracy rate and lower complication rate than ERCP-guided tissue sampling; therefore, EUS-FNA/FNB considered more useful than ERCP-guided tissue sampling for the diagnosis of pancreatic cancer [12]. The puncture routes and the target sites for EUS-FNA/FNB in hepatobiliary–pancreatic cancers are presented in Table 1.

**Table 1:** Puncture routes and target sites for EUS-FNA/FNB in hepatobiliary–pancreatic cancers

Puncture-route	HCC	IHCC	PC/IPMC/PanNEC	GBC	EHCC
Transgastric	Left lobe	Left lobe	Ph/Pb, Pb, Pb/Pt, Pt	Perihilar LN (Mets)	Perihilar LN (Mets)
	Caudate lobe	Caudate lobe	Perihilar LN (Mets)		
		Perihilar LN (Mets)	Left lobe (Mets)		
Transduodenal	Right lobe	Right lobe	Ph	GB	BD
	Around EBD	Around EBD	Right lobe (Mets)	Right lobe	Right lobe
		Perihilar LN (Mets)	Perihilar LN (Mets)	Perihilar LN (Mets)	Perihilar LN (Mets)

*EUS* endoscopic ultrasound, *FNA* fine-needle aspiration, *FNB* fine-needle biopsy, *HCC* hepatocellular carcinoma, *IHCC* intrahepatic cholangiocarcinoma, *PC* pancreatic cancer, *IPMC* intraductal papillary mucinous carcinoma, *PanNEC* pancreatic neuroendocrine carcinoma, *GBC* gallbladder carcinoma, *EHCC* extrahepatic cholangiocarcinoma, *LN* lymph nodes, *Mets* metastases, *Ph/Pb* pancreatic head/body, *Pb* pancreatic body, *Pb/Pt* pancreatic body/tail, *Pt* pancreatic tail, *Ph* pancreatic head, *EBD* extrahepatic bile duct, *GB* gallbladder, *BD* bile duct

A previous report has shown that EUS-FNB has a high diagnostic accuracy rate (85.3%) for < 20 mm pancreatic cancers (median, 16.5 mm) [13], but no study has examined the diagnostic accuracy rate of EUS-FNA/FNB for < 10 mm pancreatic cancers. Since the 5 years of survival rate of < 10 mm early pancreatic cancer is 80.4%, a high diagnostic accuracy rate of EUS-FNA/FNB for < 10 mm pancreatic cancer would be clinically useful, but only 0.8% pancreatic cancers are detected at this stage [14].

Several meta-analyses have been reported comparing EUS-FNA to EUS-FNB in solid tumors, predominantly pancreatic tumors [4, 15–21]. There are studies reporting that the diagnostic accuracies of EUS-FNA and EUS-FNB are comparable [4, 15, 17, 18, 20] and others reporting that FNB is superior [16, 19, 21]; however, most reports [4, 16, 19, 20] indicate that EUS-FNB is better in terms of diagnostic adequacy. Based on these results, the European Society of Gastrointestinal Endoscopy (ESGE) guidelines for 2021 described EUS-FNA and EUS-FNB at the same level of usefulness but recommended EUS-FNB when core tissue is needed for diagnosis, genetic profiling is required, and ROSE is not available [22]. Chen et al. performed a comparative study of EUS-FNB and EUS-FNA + ROSE and showed the non-inferiority of EUS-FNB alone regarding diagnostic accuracy [23].

### Adverse events

In Japan, the incidence of adverse events associated with EUS-FNA is 1.7% and is mainly due to hemorrhage and pancreatitis [24]. According to an overseas report [25], the incidence of adverse events associated with EUS-FNA is 0.98%, including abdominal pain, pancreatitis, hematoma, bleeding, and fever; however, the reports of serious complications are scarce, and the procedure is considered safe. In contrast, the needle tip used in EUS-FNB has a distinctive shape, which may increase adverse events, predominantly bleeding; nonetheless, several meta-analyses have reported that the incidence of adverse events is comparable between EUS-FNA and EUS-FNB [4, 15–21].

The incidence of needle tract seeding (NTS) in Japan is as low as 0.05% during EUS-FNA [24]. Similarly, a meta-analysis reported a low incidence of NTS with EUS-FNA rate of 0.3% [26]. Recently, Kawabata et al. reported a case of NTS after EUS-FNB for pancreatic cancer [27]. Nakatsubo et al. reported NTS in 2 of the 73 patients who underwent preoperative EUS-FNB for solid pancreatic tumors, with an incidence of 2.7% [28]. There is a concern that the frequency of NTS in EUS-FNB may be higher than that of EUS-FNA because a greater amount of tissue can be obtained using EUS-FNB; however, there are no comprehensive reports on the incidence of NTS. Thus, further assembly of cases is needed.

### Number of punctures

Ishigaki et al. retrospectively evaluated patients who underwent EUS-FNA or EUS-FNB for solid pancreatic tumors, reporting that the histological tissue acquisition rate in the first pass was significantly higher in the EUS-FNB than in the EUS-FNA group (87 vs. 69%, *P* = 0.007) [29]. The histological tissue acquisition rate reached a plateau after the fourth puncture in EUS-FNA, whereas it reached a plateau after the second puncture in EUS-FNB. Furthermore, the proportion of patients with a definitive diagnosis of pancreatic cancer after the first puncture was significantly higher in the EUS-FNB than in the EUS-FNA group (84 vs. 63%, *P* = 0.02).

The 2017 ESGE guidelines recommend 3–4 punctures for EUS-FNA and 2–3 punctures for EUS-FNB when ROSE cannot be performed for solid pancreatic tumors [30]. However, two randomized controlled trials (RCTs) reported that three punctures with EUS-FNA/FNB for pancreatic tumors were insufficient because the diagnostic accuracy rate did not exceed 90% [31, 32]. Zhou et al. performed an RCT to determine the optimal number of punctures for solid pancreatic tumors [33]. The cumulative diagnostic accuracy rates per number of punctures in the standard-suction group were 71.2, 85.0, 90.0, 93.3, and 95.0%, whereas those in the stylet slow-pull group were 44.8, 76.8, 87.5, 92.9, and 94.6%. The authors reported that at least three and four punctures should be performed in the standard-suction and slow-pull groups, respectively. Likewise, several meta-analyses reported that the number of punctures required to confirm the diagnosis was significantly lower with EUS-FNB than with EUS-FNA [4, 15, 16, 19–21]. However, a prospective study with a large number of cases is needed to determine the optimal number of punctures with EUS-FNA/FNB.

## Genomic analysis of pancreatic cancers using EUS-FNA/FNB specimens

**Fig. 1:**
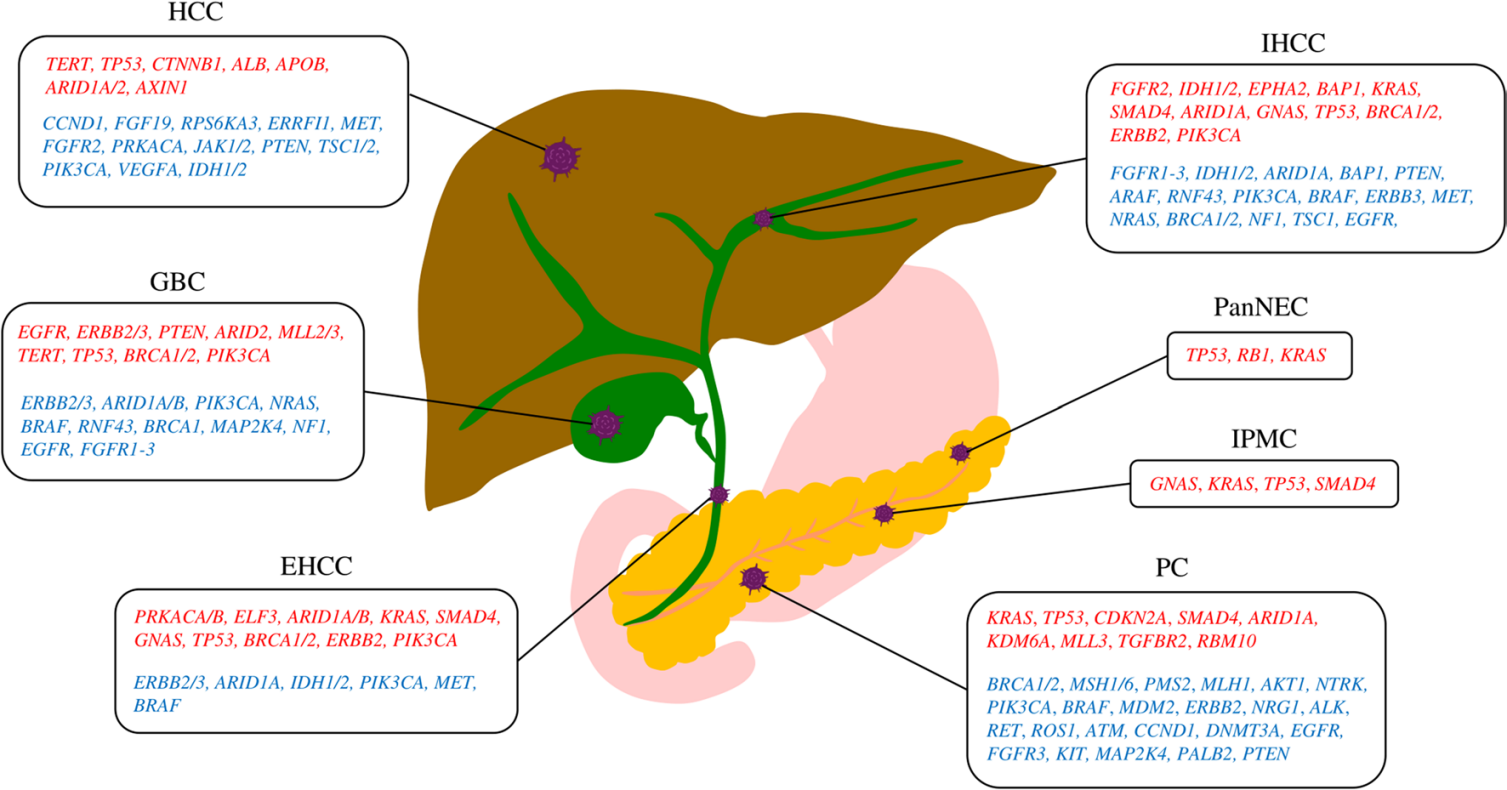
Representative gene mutations in hepatobiliary–pancreatic cancers. Driver and actionable mutations are listed in red and blue, respectively. *HCC* hepatocellular carcinoma, *IHCC* intrahepatic cholangiocarcinoma, *GBC* gallbladder carcinoma, *EHCC* extrahepatic cholangiocarcinoma, *PanNEC* pancreatic neuroendocrine carcinoma, *IPMC* intra-ductal papillary mucinous carcinoma, *PC* pancreatic cancer

The mutational landscape of pancreatic ductal adenocarcinoma (PDAC) is dominated by driver mutations in *KRAS*, *TP53*, *CDKN2A*, and *SMAD4*, which occur alone or in combination in > 95% cases, whereas mutations in various other genes, including *ATM*, *BRCA1*, *ARID1A*, *KDM6A*, *MLL3*, *TGFBR2*, *RBM10*, and *BCORL1*, are found in < 10% cancers (Fig. 1) [34–38]. Many studies on *KRAS* have reported genetic analyses using EUS-FNA. In a meta-analysis, the diagnostic performance of *KRAS* mutations in EUS-FNA specimens was reported to have a sensitivity and specificity of 79 and 94%, respectively [39]. An 83–100% concordance rate was found when abnormalities, such as *KRAS*, *TP53*, and *SMAD4*, were compared between EUS-FNA and surgically resected specimens [40, 41].

Intra-ductal papillary mucinous carcinoma (IPMC) accounts for 10% pancreatic cancers of ductal origin. Compared to conventional PDAC, IPMC has specific clinical characteristics and favorable pathological features. The main genomic alterations in IPMC include *GNAS* and *KARS* (Fig. 1) [42–44]. Additionally, intra-ductal papillary mucinous neoplasm (IPMN) progress to invasive carcinomas with an accumulation of abnormalities in *TP53* and *SMAD4* (Fig. 1) [45]. Pancreatic neuroendocrine carcinomas (PanNECs) have high-grade, carcinoma-like nuclear features and characteristically exhibit aggressive clinical behavior, frequent metastases, and poor survival, unlike pancreatic neuroendocrine tumors (panNETs) [46]. The molecular profile of PanNECs is characterized by *TP53*, *RB1*, and *KRAS* mutations, which are the key drivers of Pan-NEC (Fig. 1) [46, 47].

Several sequencing analyses of pancreatic cancers using EUS-FNA/FNB specimens have recently been reported (Table 2) [35–37, 40, 41, 48–62]. The success rate of sequencing analysis using EUS-FNA/FNB specimens is reported to be 42–100%, although there is considerable variation among reports. We performed CGP analysis of solid-pseudopapillary neoplasm (SPN) of the pancreas using EUS-FNA specimens, reporting that *CTNNB1* mutations were detected in all cases. Thus, CGP analysis of EUS-FNA specimens may be useful for low-grade malignant tumors, such as SPN [63]. The success rate of sequencing analysis of pancreatic cancers using surgically resected specimens has been reported to be 90–100%, and the success rate of sequencing analyses using EUS-FNA/FNB specimens is low [51, 64]. Insufficient sample volumes of tissue and number of cells, as well as degradation of DNA quality, are considered reasons for the failure of sequencing analysis [48, 65]. In a study comparing tissue sampling methods between EUS-FNA and EUS-FNB, EUS-FNB achieved a higher proportion of diagnostic adequacy than EUS-FNA (90.9 vs. 66.9%, *P* = 0.02). In multivariate analysis, only EUS-FNB (OR: 4.95, 95% CI 1.11–22.05, *P* = 0.04) was identified as an independent factor contributing to the success of the genomic analysis [37]. A meta-analysis comparing Franseen and fork-tip needles for EUS-FNB showed a high core tissue collection rate (> 90%) for both needles [66].

**Table 2:** Sequencing analysis of pancreatic cancers using EUS-FNA/FNB

Year	References	Patientnumber	Cancertype	Samplingmethods	Analysismethods	Analysistarget	Success rate ofsequencing analysis	Detection rate ofactionable mutations
2013	Young et al. [41]	23	PDCA (18) Others (5)	FNA	Targeted seq	287 genes	100%	ND
2016	Valero et al. [55]	17	ND	FNA	Targeted seq with MB	409 genes	89.5%	23.5%
2016	Rodriguez et al. [56]	23	PDAC (15) Others (8)	FNA	RNA seq	85 genes	71.9%	ND
2016	Kameta et al. [35]	38	PDAC (27) Others (11)	FNA	Targeted seq	50 genes	100%	ND
2016	Gleeson et al. [41]	47	ND	FNA	Targeted seq	160 genes	61.7%	0%
2017	Lowery et al. [54]	52	PDAC (52)	FNA/FNB	Targeted seq	410 genes	ND	ND
2018	Larson et al. [48]	61	ND	FNA/FNB	Targeted seq	ND	67.2%	ND
2019	Elhanafi et al. [37]	167	ND	FNA/FNB	Targeted seq	47 genes	70.1%	ND
2019	Dreyer et al. [36]	41	PDAC (36) Others (5)	FNB	WGS	ND	72%	16%
2020	Ishizawa et al. [52]	26	PDAC (26)	FNA	Targeted seq	409 genes	100%	ND
2020	Park et al. [53]	190	PDAC (190)	FNA/FNB	Targeted seq	83 genes	57.4%	ND
2021	Kandel et al. [49]	50	PDAC (37) Others (13)	FNA/FNB	Targeted seq	ND	92%	ND
2021	Takano et al. [57]	58	PDAC (58)	FNA/FNB	Targeted seq with MB	50 genes	94.8%	22.4%
2021	Habib et al. [58]	59	PDAC (56) Others (3)	FNA	Targeted seq	9 genes	ND	ND
2021	Semaan et al. [59]	23	PDAC (23)	FNA	EpCAM-pulldown combinedwith MB WES	ND	100%	21.7%
2021	Carrara et al. [60]	33	PDAC (33)	FNB	Targeted seq	21 genes	97%	ND
2021	Kondo et al. [51]	22	ND	FNA	Targeted seq	324 genes	68.2%	ND
2022	Gan [50]	26	PDAC (26)	FNA/FNB	Targeted seq	ND	42–81%	ND
2022	Hisada et al. [61]	33	PDAC (31) Others (2)	FNB	Targeted seq	124 genes	57.1%	33.3%
2023	Ikeda et al. [62]	30	PDAC (30)	FNA/FNB	Targeted seq	124 genes	100%	ND

*ND* not described, *EUS* endoscopic ultrasound, *FNA* fine-needle aspiration, *FNB* fine-needle biopsy, *PDAC* pancreatic ductal adenocarcinoma, *seq* sequence, *WGS* whole-genome sequence, *MB* molecular barcodes, *WES* whole exome sequence

Park et al. retrospectively examined factors related to the success of CGP analysis in 190 patients who underwent EUS-FNA/FNB for pancreatic tumors, reporting that only the external diameter of the puncture needle was a significant factor related to the success of CGP analysis in a multivariate analysis [53]. The success rate of CGP analysis was significantly lower for the 25-gage needles than for the 19/22-gage needles (38.8 vs. 60.9%, *P* = 0.003) [53]. Kandel et al. examined the proportion of fulfillment of the requirement for CGP analysis with a single puncture, comparing 25- and 19/22-gage needles in patients who underwent EUS-FNA/FNB for pancreatic tumors [49]. Additionally, 78% of patients with 19/22-gage needles fulfilled the requirement for CGP analysis, whereas the rate was as low as 14% with 25-gage needles. Based on these reports, the success rate of CGP analysis may be less with 25-gage puncture needles.

There are two reports of NOP analysis of specimens collected by EUS-FNA/FNB for pancreatic cancers in Japan. Hisada et al. performed an NOP analysis on 63.6% (21/33) of specimens collected by EUS-FNB from pancreatic cancers that met NOP analysis suitability criteria (tumor cell content ≥ 20% and tissue size ≥ 4 mm) and reported that the success rate of NOP analysis was 57.1% (12/21) [61]. In a similar study, Ikeda et al. reported that NOP analysis suitability criteria were met in 39.2% (60/153) of specimens collected by EUS-FNA/FNB from pancreatic cancers, of which 30 cases underwent NOP analysis, with a success rate of 100% (30/30) [62]. It is considered important to meet suitability criteria to increase the success rate of NOP analysis, and multivariate analysis has identified the use of 19-gage needles and EUS-FNB as contributing factors to NOP analysis suitability criteria [62].

As mentioned above, 3–4 and 2–3 punctures are recommended for pathological diagnosis of pancreatic tumors using EUS-FNA and EUS-FNB, respectively. However, the optimal number of punctures for sequencing analysis has not been clarified. According to previous reports, it is possible to collect sufficient samples for sequencing analysis with 1–3 punctures of EUS-FNA and FNB [49, 67]. Nonetheless, there are cases in which the sample volume is low even if the number of punctures is increased, and the variation among cases is considerable.

In contrast, pancreatic cancer is a typical low-cellularity tumor with a high stromal component, while its tumor component content is approximately 5–20% [68]. Therefore, pancreatic cancer is considered a challenging tumor for sequencing analysis. The success rate of sequencing analysis using EUS-FNA/FNB specimens tends to be lower than that of other gastrointestinal cancers [69, 70].

**Fig. 2:**
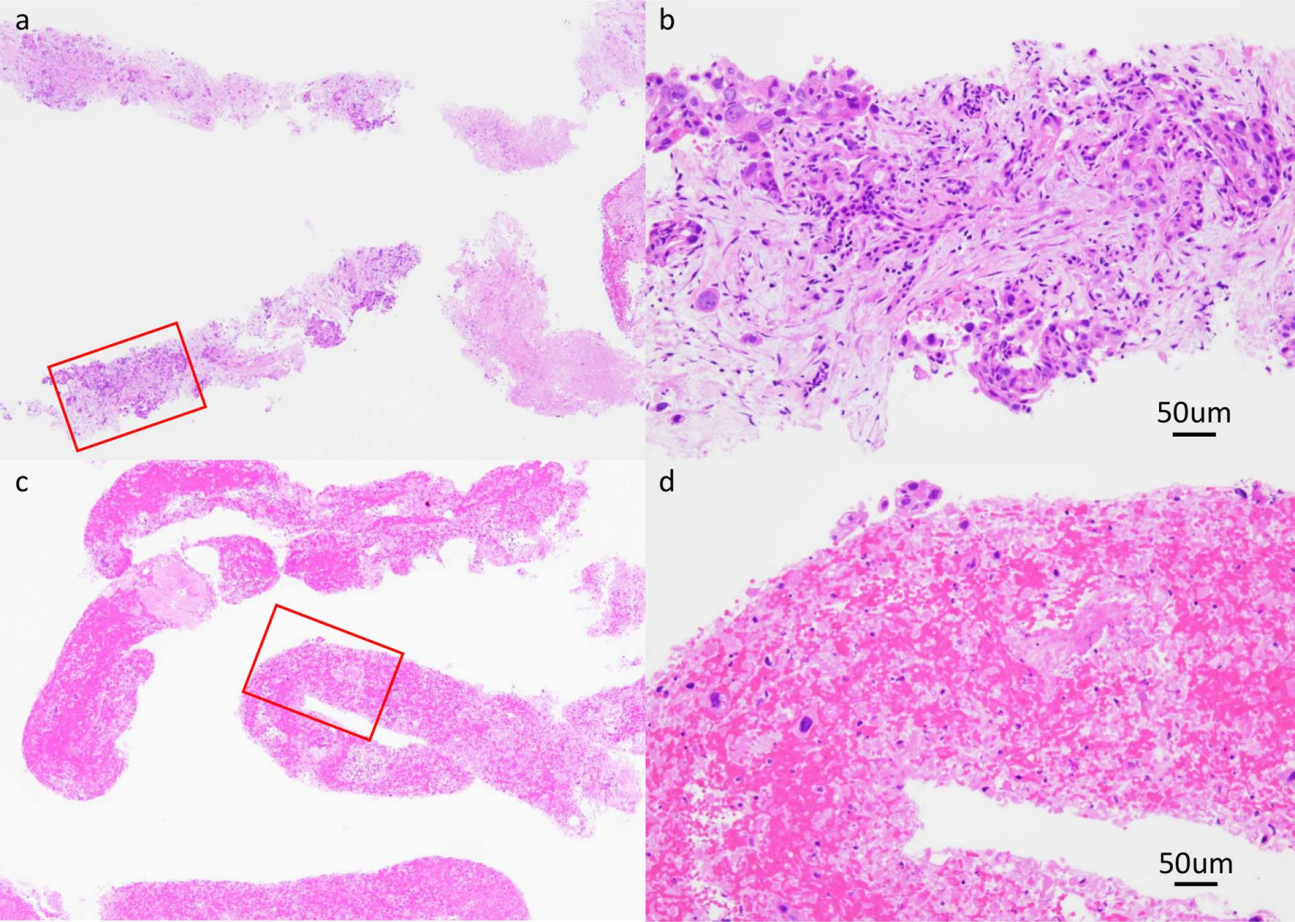
Comparison of the representative EUS-FNB and EUS-FNA specimens in the same patient with pancreatic cancer. A 22-mm pancreatic cancer lesion was punctured with a 22-gage FNB and FNA needle. (**a**: Left upper) The EUS-FNB specimen obtained using a 22-gage FNB tri-tip core needle revealed multiple histological tissues. (**b**: Right upper) The tissue microfragment with intact tissue architecture was diagnosed as moderately differentiated pancreatic adenocarcinoma. These FNB specimens contributed to the suitability of CGP analysis. (**c**: Left bottom) The EUS-FNA specimen obtained using a 22-gage FNA lancet needle did not include tissue microfragments. (**d**: Left bottom) Most characteristic specimens showed blood clots. A sufficient number of tumor cells was not observed. Tumor cellularity of the specimens was 10% (88/892 cells) and 50% (618/1247 cells) for EUS-FNA and EUS-FNB, respectively. Very little tissue was collected by EUS-FNA, and most of the nucleated cells were neutrophils in the peripheral blood. Although pancreatic cancer was diagnosed, we speculated that CGP analysis of these specimens was impossible and/or unsuitable. EUS-FNA specimens sometimes include tissues, making it possible to perform CGP analyses. *EUS* endscopic ultrasound, *FNA* fine-needle aspiration, *FNB* fine-needle biopsy, *CGP* comprehensive genomic profiling

When biopsy specimens are used for the analysis of genetic abnormalities, the quality of DNA and RNA, and the collection of a sufficient tumor volume are required. Representative specimens from the same patients with pancreatic cancer who underwent EUS-FNA and EUS-FNB are shown (Fig. 2). EUS-FNB specimens revealed multiple histological tissues and tissue microfragments, whereas the EUS-FNA specimen did not include tissue microfragments or sufficient tumor cells. In a study comparing the sample quality obtained by EUS-FNA and EUS-FNB for pancreatic cancers, a significantly higher sample volume [71], cellularity [72–74], and DNA/RNA yield [49, 67] were obtained with EUS-FNB than those with EUS-FNA. Kandel et al. reported that the median tumor cellularity of the specimens was 40% and 10%, and the DNA concentration was 5.93 μg/ml and 3.37 μg/ml for EUS-FNB and EUS-FNA, respectively [49]. In CGP analysis in Japan (NOP and F1CDx), a minimum of 20% tumor cellularity is recommended; therefore, EUS-FNB is preferred over EUS-FNA for CGP analysis. In addition, it has been reported that the success rate of sequencing analysis is lower for formalin-fixed paraffin-embedded (FFPE) samples than for fresh tumor tissues due to the susceptibility to DNA quality degradation. The success rate of sequencing analysis using FFPE specimens was 84.8%, whereas that using fresh tumor tissue was significantly higher, at 97.4% (*P* < 0.05) [75]. Therefore, the specimen type should be carefully considered [76].

As mentioned above, pancreatic cancer has few gene alterations other than those on major driver genes, such as *KRAS*, *TP53*, *CDKN2A*, and *SMAD4*; however, genome-matched therapy based on CGP analysis has been reported to prolong the prognosis of patinets with pancreatic cancer [77]. Several reports have identified actionable mutations in pancreatic cancer using sequencing analysis, increasing treatment options (1–26%) (Fig. 1) [54, 77–81]. Moreover, a study of CGP analysis in several patients with pancreatic cancer revealed that gene abnormalities, such as *BRCA2*, *BRAF*, *ERBB2*, *CDK12*, *PIK3CA*, *FGFR2*, and *EGFR*, are more frequent in patients with pancreatic cancer lacking *KRAS* mutations; therefore, patients with pancreatic cancer lacking *KRAS* mutations should undergo CGP analysis [82]. In addition, CGP analysis can be performed for not only pancreatic cancer, but also low-grade malignant tumors such as SPN or pancreatic neuroendocrine tumors, which may lead to genome-matched therapy. Therefore, the importance of CGP analysis is expected to increase in future. To improve the success rate of CGP analysis using EUS-FNA/FNB specimens, it is necessary to prospectively study a large number of cases, including the selection of the puncture needle, number of punctures, aspiration method, and specimen type.

## Genomic analysis of biliary tract cancers using EUS-FNA/FNB specimens

The types of driver gene mutations in biliary tract cancers vary greatly depending on the cancer anatomical classification, including *FGFR2*, *IDH1/2*, *EPHA2*, *BAP1*, *KRAS*, *SMAD4*, *ARID1A*, *GNAS*, *TP53*, *BRCA1/2*, *ERBB2*, and *PIK3CA* in intrahepatic cholangiocarcinoma (IHCC); *PRKACA/B*, *ELF3*, *ARID1A/B*, *KRAS*, *SMAD4*, *GNAS*, *TP53*, *BRCA1/2*, *ERBB2*, and *PIK3CA* in extrahepatic cholangiocarcinoma (EHCC); and *EGFR*, *ERBB2/3*, *PTEN*, *ARID2*, *MLL2/3*, *TERT*, *TP53*, *BRCA1/2*, and *PIK3CA* in gallbladder carcinoma (GBC) [83–85] (Fig. 1). Biliary tract cancers do not have a particularly high frequency mutations, such as *KRAS* in pancreatic cancer, but rather a high presence of relatively low-frequency mutations. Furthermore, approximately 40% of biliary tract cancers have actionable mutations that can serve as therapeutic targets (Fig. 1) [84–95]. The National Cancer Center Network guidelines list eight druggable markers in biliary tract cancer (*NTRK* fusion, *MSI-H*, *TMB-H*, *BRAF*, *V600E*, *FGFR2* fusions/rearrangement, *IDH1* mutations, *RET* fusion, and *HER2* overexpression) and their corresponding therapeutic agents [96].

In the reports regarding tissue sampling, when EUS-FNA was compared with forceps biopsy and brush cytology during ERCP for malignant biliary stricture, including biliary tract cancers, EUS-FNA had higher sensitivity, specificity, and diagnostic accuracy than forceps biopsy and brush cytology during ERCP, being more useful [97, 98]. Moreover, in a study comparing peroral cholangioscopy-guided forceps biopsy (POC-FB) and EUS-FNA/FNB for malignant biliary strictures, POC-FB was recommended for proximal and intrinsic strictures, whereas EUS-FNA/FNB was recommended for distal and extrinsic strictures, with a high diagnostic accuracy rate [99].

Several sequencing analyses of biliary tract cancers using EUS-FNA/FNB specimens have recently been reported (Table 3) [40, 100–103]. In 2019, Hirata et al. performed EUS-FNA in 21 cases of biliary tract cancer and reported that CGP analysis was possible in 20 cases (95.2%). In addition, actionable mutations were identified in 7 of them for the first time [100]. Kai et al. performed EUS-FNA in 12 patients with advanced or postoperative recurrent biliary tract cancer, reporting that CGP analysis could be performed in all patients [101]. Maruki et al. performed FISH + targeted RNA sequencing analysis of FFPE specimens collected by EUS-FNA in 26 patients with advanced or postoperative recurrent biliary tract cancer to determine whether *FGFR2* rearrangement was present, finding mutations in two patients (7.7%) [102]. As actionable mutations have been identified in biliary tract cancers by CGP analysis using EUS-FNA/FNB specimens, the importance of CGP analysis is likely to increase, similar to pancreatic cancer.

**Table 3:** Sequencing analysis of biliary tract cancers using EUS-FNA/FNB

Year	References	Patient number	Cancer type	Sampling methods	Analysis methods	Analysis target	Success rate of sequencing analysis	Detection rate of actionable mutations
2016	Gleeson et al. [40]	4	AC (4)	FNA	Targeted seq	160 genes	ND	ND
2017	Choi et al. [103]	13	IHCC (7) GBC (5) AC (1)	FNB	PNA-PCR Targeted seq	*KRAS*	ND	ND
2019	Hirata et al. [100]	21	IHCC (6) EHCC (3) GBC (12)	FNA	Targeted seq	50 genes	95.2%	33%
2021	Kai et al. [101]	12	IHCC (3) EHCC (2) GBC (7)	FNA	Targeted seq	MSI	100%	0%
2021	Maruki et al. [102]	26	ND	FNA	FISH + targeted RNA seq	*FGFR2*	ND	7.7%

*ND* not described, *EUS* endoscopic ultrasound, *FNA* fine-needle aspiration, *FNB* fine-needle biopsy, *seq* sequence, *PNA* peptide nucleic acid, *PCR* polymerase chain reaction, *FISH* fluorescent in situ hybridization, *AC* ampullary cancer, *IHCC* intrahepatic cholangiocarcinoma, *GBC* gallbladder carcinoma, *EHCC* extrahepatic cholangiocarcinoma, *MSI* microsatellite instability

EUS-FNA/FNB- or ERCP-guided tissue sampling is performed for IHCC and EHCC (particularly localized bile duct carcinoma), whereas ERCP-guided tissue sampling is performed for EHCC (particularly diffuse sclerosing bile duct carcinoma) [104]. In particular, CGP analysis of malignant bile duct strictures using ERCP-guided tissue sampling specimens have reported high sensitivity and specificity (72% and 100%, respectively) [105], suggesting that CGP analysis using ERCP-based tissue sampling specimens may be feasible if there is a sufficient sample volume. However, if the lesion is located outside the bile duct, such as in lymph nodes or liver metastases, EUS-FNA/FNB can be used for tissue sampling, and the tissue sampling strategy should be considered according to the lesion location [106]. Regarding GBC, ERCP-guided tissue sampling is often difficult to selectively cannulate the gallbladder duct and associated with the incidental perforation of cystic duct in addition to post-ERCP pancreatitis. Therefore, EUS-FNA/FNB is the first choice for puncturing the biliary tract via a non-luminal route [106].

Alternative methods for tissue fixation instead of FFPE include the use of frozen specimens or storing specimens in RNA later (Life Technologies, Carlsbad, CA). When frozen specimens are used for CGP analysis, additional collections are required in addition to those for diagnostic purposes at the time of EUS-FNA/FNB, but it has been reported that frozen specimens have good nucleic acid quality and can be stored for long periods [107]. Hirata et al. reported that rapid tissue preservation in RNA later and immediate refrigeration after EUS-FNA for biliary tract cancers can preserve DNA quality by preventing fragmentation and chemical modification, which are common in FFPE, resulting in a high success rate for CGP analysis [100].

In addition, there are methods using cellular samples and digital PCR to obtain a high success rate for CGP analysis even from small EUS-FNA/FNB specimens. In many cases, EUS-FNA/FNB cannot collect sufficient tissue, and only cellular specimens can be obtained. Cellular specimen processing methods include cell block, smear, and liquefied specimen cytology, all of which are capable of extracting high-quality nucleic acids for genomic analysis [108–110]. Digital PCR, the third-generation PCR, is limited in the number of genes that can be analyzed, but it is reported to be capable of analyzing genes even in very small amounts [111]. Although there are some reports of CGP analyses using frozen specimens [35, 53, 55, 60], cellular specimens [37, 40, 52], and digital PCR [112] collected by EUS-FNA/FNB for pancreatic cancers, therefore, case accumulation of CGP analysis using these methods for biliary tract cancers is needed.

## Genomic analysis of liver cancers using EUS-FNA/FNB specimens

A meta-analysis summarizing reports of whole-genome analyses of 1,340 cases of hepatocellular carcinoma (HCC) identified a large number of driver genes, among which *TERT* (> 50%), *TP53* (29.1%), *CTNNB1* (28.6%), *ALB* (10.2%), *APOB* (9.8%), *ARID1A* (8.8%), *ARID2* (8.2%), and *AXIN1* (7.5%) gene mutations were highly prevalent [113] (Fig. 1). Approximately 25% HCC harbors potentially actionable mutations, but these mutations have not been translated into the clinical practice yet (Fig. 1) [114, 115]. Moreover, the mutational drivers of HCC, such as *TERT*, *TP53*, and *CTNNB1*, are un-druggable [114].

Because of the established percutaneous liver biopsy to tissue sampling in liver tumors, the 2021 ESGE guidelines weakly recommend EUS-guided biopsy for liver tumors, recommending it only under exceptional circumstances, such as for anatomical issues and upon failure of percutaneous biopsy [22]. Recently, Ichim et al. reported the usefulness of EUS-FNA for liver tumors, which was performed in 30 cases of liver tumors where percutaneous liver biopsy was difficult due to a small tumor diameter or distance from the puncture site; diagnostic adequacy was obtained in 29 of these cases (97%) [116].

Moreover, a study comparing the diagnostic performance of percutaneous liver biopsy and EUS-FNA for liver tumors reported that the sensitivity, specificity, and diagnostic accuracy rate were comparable. However, complications were significantly low with EUS-FNA (17 vs. 2%, *P* < 0.01) [106]. The reasons for less complications with EUS-FNA/FNB are: the puncture needle used in EUS-FNA/FNB is smaller in diameter than that used in percutaneous liver biopsy (19–25 gage vs. 16–18 gage), EUS has a high spatial resolution and can avoid small vessels, and EUS-FNA/FNB is unaffected by subcutaneous fat or the intestinal tract, unlike percutaneous liver biopsy [117].

Several studies have reported that percutaneous liver biopsy can be performed for CGP analysis in primary or metastatic liver cancers [75, 118]. Eso et al. [75] and Ozeki et al. [118] reported that the success rate of CGP analysis was 100% (22/22) and 84.9% (62/73), respectively. Moreover, several studies have been reported on sequencing analysis of liver cancers using EUS-FNA/FNB specimens (Table 4) [101, 103]. Choi et al. performed EUS-FNB for solid liver cancers in the left lobe [103]. In this study, 12 patients had primary liver cancer (including four HCCs and seven IHCCs), and 16 had metastatic liver cancer (including seven pancreatic cancers five GBCs). CGP analysis was performed on 16 of these cases (57%), detecting *KRAS* mutations. Kai et al. also reported CGP analysis using EUS-FNA specimens for metastatic liver cancer [101]. These reports [101, 103] suggest that in cases where it is difficult to obtain tissue samples from the primary lesion for reasons, such as anatomical issues and small tumor size, EUS-FNA/FNB may be useful for obtaining tissue samples from liver tumors if there are metastatic lesions in the liver.

**Table 4:** Sequencing analysis of liver cancers using EUS-FNA/FNB

Year	References	Patient number	Cancer type	Sampling methods	Analysis methods	Analysis target	Success rate of sequencing analysis	Detection rate of actionable mutations
2017	Choi et al. [103]	28	HCC (4)	FNB	PNA-PCR	*KRAS*	96.4%	14.3%
			IHCC (7)		Targeted seq		57%	25%
			MLC (16)					
			Others (1)					
2021	Kai et al. [101]	1	IHCC (1)	FNA	Targted seq	MSI	100%	0%

*EUS* endoscopic ultrasound, *FNA* fine-needle aspiration, *FNB* fine-needle biopsy, *seq* sequence*, PNA* peptide nucleic acid, *PCR* polymerase chain reaction, *HCC* hepatocellular carcinoma, *IHCC* intrahepatic cholangiocarcinoma, *MLC* metastatic liver cancer, *MSI* microsatellite instability

As mentioned above, few actionable mutations lead to the treatment of HCC; therefore, few therapeutic agents can lead to genome-matched therapy currently, even if the tissue is obtained by EUS-FNA/FNB. Further progress in the genomic analysis of HCC and its relationship with clinical information, such as the efficacy of molecular-targeted drugs and immune checkpoint inhibitors, based on big data will lead to the expansion of the indications of existing molecular-targeted drugs for HCC. However, CGP analysis using EUS-FNA/FNB specimens is useful for metastatic liver cancer, particularly metastatic pancreatic or biliary tract cancer, because actionable mutations that can lead to genome-matched therapy are recognized.

## Conclusion

CGP based on NGS analysis is often performed on surgically resected specimens in daily practice. However, surgical tissue sampling for CGP analysis is difficult for patients with un-resectable pancreatic and biliary tract cancers. Tissue sampling using EUS-FNA/FNB allows CGP analysis in inoperable patients with pancreatic and biliary tract cancers, leading to genome-matched therapy. Therefore, tissue sampling using EUS-FNA/FNB is clinically significant for pancreatic and biliary tract cancers. Notably, genome-matched therapy based on CGP analysis has been shown to improve the prognosis of pancreatic cancer patients. However, the success rate of CGP analysis in EUS-FNA/FNB specimens compared to that in surgically resected specimens is currently not clinically sufficient. For many patients with pancreatic and biliary tract cancers to benefit from CGP analysis, it is essential to accumulate evidence through prospective studies of a large number of cases, including the selection of puncture needle, number of punctures, aspiration method in EUS-FNA/FNB, and specimen type, to improve the success rate of CGP analysis.
